# High positive end-expiratory pressure: only a dam against oedema formation?

**DOI:** 10.1186/cc12810

**Published:** 2013-07-11

**Authors:** Alessandro Protti, Davide T Andreis, Giacomo E Iapichino, Massimo Monti, Beatrice Comini, Marta Milesi, Loredana Zani, Stefano Gatti, Luciano Lombardi, Luciano Gattinoni

**Affiliations:** 1 Dipartimento di Anestesia, Rianimazione (Intensiva e Subintensiva) e Terapia del Dolore, Fondazione IRCCS Ca' Granda - Ospedale Maggiore Policlinico, via Francesco Sforza, 35, 20122 Milan, Italy; 2 Dipartimento di Fisiopatologia Medico-Chirurgica e dei Trapianti, Università degli Studi di Milano, Via Francesco Sforza, 35, 20122 Milan, Italy; 3 Centro di Ricerche Chirurgiche Precliniche, Fondazione IRCCS Ca' Granda - Ospedale Maggiore Policlinico, Università degli Studi di Milano, Via Francesco Sforza, 35, 20122 Milan Italy; 4 Dipartimento di Radiologia, Fondazione IRCCS Ca' Granda - Ospedale Maggiore Policlinico, via Francesco Sforza, 35, 20122 Milan, Italy

## Abstract

**Introduction:**

Healthy piglets ventilated with no positive end-expiratory pressure (PEEP) and with tidal volume (V_T_) close to inspiratory capacity (IC) develop fatal pulmonary oedema within 36 h. In contrast, those ventilated with high PEEP and low V_T_, resulting in the same volume of gas inflated (close to IC), do not. If the real threat to the blood-gas barrier is lung overinflation, then a similar damage will occur with the two settings. If PEEP only hydrostatically counteracts fluid filtration, then its removal will lead to oedema formation, thus revealing the deleterious effects of overinflation.

**Methods:**

Following baseline lung computed tomography (CT), five healthy piglets were ventilated with high PEEP (volume of gas around 75% of IC) and low V_T _(25% of IC) for 36 h. PEEP was then suddenly zeroed and low V_T _was maintained for 18 h. Oedema was diagnosed if final lung weight (measured on a balance following autopsy) exceeded the initial one (CT).

**Results:**

Animals were ventilated with PEEP 18 ± 1 cmH_2_O (volume of gas 875 ± 178 ml, 89 ± 7% of IC) and V_T _213 ± 10 ml (22 ± 5% of IC) for the first 36 h, and with no PEEP and V_T _213 ± 10 ml for the last 18 h. On average, final lung weight was not higher, and actually it was even lower, than the initial one (284 ± 62 vs. 347 ± 36 g; *P *= 0.01).

**Conclusions:**

High PEEP (and low V_T_) do not merely impede fluid extravasation but rather preserve the integrity of the blood-gas barrier in healthy lungs.

## Introduction

Mechanical ventilation is a pivotal therapy for respiratory failure, although overinflation may injure the lung [1-3].

We have recently shown that healthy piglets ventilated with no positive end-expiratory pressure (PEEP) and with tidal volume (V_T_) close to inspiratory capacity (IC) die with inflammatory pulmonary oedema within 36 h. In contrast, those ventilated with high PEEP (≥18 cmH_2_O; volume of gas around 75% of IC) and low V_T _(25% of IC) survive with normal lungs for 54 h [4].

Inflammatory pulmonary oedema develops when lung capillary transmural (internal minus external) pressure drives excessive fluid filtration through a disrupted, highly permeable, blood-gas barrier [5]. As a result, exudates accumulate in the extravascular space and lung weight increases [6,7]. If overinflation *per se *is the real threat to the blood-gas barrier [8], then ventilation with large V_T _alone (100% of IC) will be as harmful as ventilation with high PEEP (75% of IC) and low V_T _(25% of IC). In fact, in both cases, the volume of gas globally inflated will be equal to IC. However, high PEEP (and low V_T_) will possibly impede extravasation by lowering venous return, cardiac output and pulmonary capillary inflow (and pressure) while increasing extravascular pressure [9,10]. Oedema will then not develop even if the blood-gas barrier loses its integrity.

To test this hypothesis, we ventilated healthy piglets with high PEEP (and low V_T_) (as above) and then suddenly removed PEEP to allow free extravasation through a possibly disrupted blood-gas barrier. Lung weight was expected to increase as oedema formation was no longer impeded.

## Materials and methods

The study complied with international recommendations [11] and was approved by the Italian Ministry of Health (protocol number 01/10).

Five healthy, sedated and paralysed piglets (21 ± 1 kg) were surgically prepared in supine position. They were then turned prone and ventilated with no PEEP and with a V_T _of 10 ml/kg of body weight (Engström Carestation, GE Healthcare; Madison, WI, USA). Lung computed tomography (CT) was taken at 0 cmH_2_O (functional residual capacity, FRC), around 18 cmH_2_O (the level of PEEP planned to be used) (see below) and 45 cmH_2_O (arbitrarily defined as total lung capacity, TLC) of airway pressure. Lung gas volumes and weight were measured by quantitative analysis [12].

**Figure 1:**
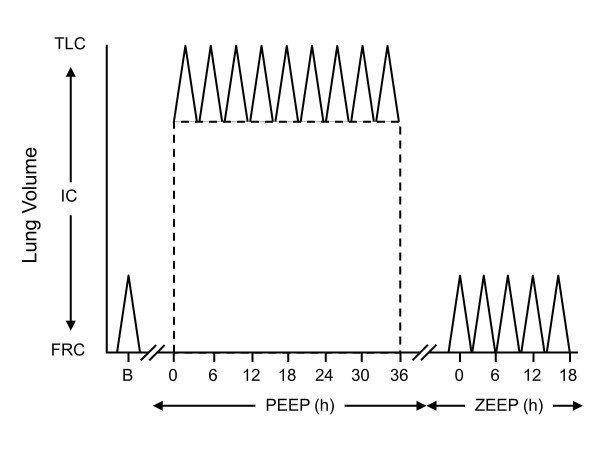
**Study design**. Initial lung weights were measured by CT. Animals were then ventilated with high PEEP (volume of gas around 75% of IC; dotted line) and low VT (25% of IC; spikes) for 36 h (PEEP phase). Thereafter, PEEP was suddenly zeroed and low VT maintained for 18 additional h (ZEEP phase). Animals were then sacrificed and final lung weights were measured on a balance. B, baseline; CT, computed tomography; FRC, functional residual capacity; IC, inspiratory capacity; PEEP, positive end-expiratory pressure; TLC, total lung capacity; V_T_, tidal volume; ZEEP, zero end-expiratory pressure..

Following this pre-study period, PEEP was set around 18 cmH_2_O while V_T _was kept constant (PEEP phase). Based on a previous study [4], we expected that the volume of gas so inflated in the form of PEEP would have been around 75% and V_T _around 25% of IC (TLC minus FRC). Therefore, the volume of gas inflated by end of inspiration (volume of gas due to PEEP plus V_T_) was predicted to be equal to IC and end-inspiratory lung volume, including FRC, to approach TLC. This setting was kept constant for 36 h (PEEP phase). PEEP was then suddenly zeroed and V_T _was maintained for 18 h (ZEEP phase) (Figure 1).

Results of the lung CT scan analysis became available once the study had already started. They did not influence the setting described above but permitted exact computation of volumes of gas effectively inflated (as PEEP and V_T_) in relation to lung capacities.

During the entire 54-h study period the animals were kept prone, in 10° Trendelemburg position [13]. Respiratory rate was 15 breaths per minute, inspiratory-to-expiratory time ratio 1:2 (1:3 if intrinsic PEEP developed) and inspired oxygen fraction was 0.50. Tracheal suctioning and recruitment manoeuvres were only performed before applying PEEP and after its removal. Use of normal saline and norepinephrine was standardised to try to maintain mean arterial pressure above 60 mmHg. Respiratory system and lung mechanics, blood gas analysis and haemodynamics (including cardiac output by thermodilution) were assessed every 6 h. Data were also collected during the pre-study period (baseline) and soon after PEEP removal (time 0 of the ZEEP phase). Transpulmonary pressure was computed as (end-inspiratory airway pressure) - [(end-inspiratory oesophageal pressure) - (oesophageal pressure at 0 cmH_2_O of airway pressure)]). Transmural pulmonary arterial and pulmonary artery occlusion pressures were computed as (end-expiratory intravascular pressures) - [(end-expiratory oesophageal pressure) - (oesophageal pressure at 0 cmH_2_O of airway pressure)]) [14]. Urinary output was recorded every hour and water balance was computed as the difference between saline infusion and urinary output.

At the end of the study, animals were sacrificed (potassium chloride, 40 mEq intravenous) and exsanguinated. Lungs were excised and weighted on a balance. This (final) lung weight was compared with the initial one, measured on pre-study CT taken at 0 cmH_2_O of airway pressure (the degree of agreement between these two methods for measuring lung weight was assessed in five other piglets, as reported in Additional file 1). Pulmonary oedema was diagnosed if lung weight had increased across the entire study period. The right lung was used for the calculation of wet-to-dry weight ratio and blind histological examination [15]. Twelve piglets uneventfully ventilated for 54 h with no PEEP and low V_T _(as part of a previous study [3]) that did not develop pulmonary oedema were used as controls.

### Statistical analysis

Numerical data are reported as mean ± standard deviation. Based on distribution (Shapiro-Wilk test), they were analysed using Student's (paired) *t *test or Mann-Whitney rank sum test, one-way repeated measures analysis of variance (RM ANOVA) or RM ANOVA on ranks. Post hoc comparisons were done using the Holm-Sidak or Dunn's method. Categorical data are reported as median (interquartile range) and were analysed using the Mann-Whitney rank sum test. A *P *value <0.05 was considered significant (SigmaPlot 11.0, Jandel Scientific Software; San Jose, CA, USA).

For more information on methods, please refer to Additional file 2.

## Results

Animals were ventilated with PEEP 18 ± 1 cmH_2_O and V_T _213 ± 10 ml for the first 36 h (PEEP phase) and with no PEEP and V_T _213 ± 10 ml for the last 18 h (ZEEP phase).

On baseline lung CT (Additional file 3), application of PEEP 18 ± 1 cmH_2_O resulted in inflation of 875 ± 178 ml of gas, equal to 89 ± 7% of estimated IC (988 ± 176 ml). V_T _was 22 ± 5% of estimated IC. The volume of gas inflated by end of inspiration during the PEEP phase (volume of gas due to PEEP plus V_T_) was slightly higher than estimated IC (1088 ± 177 vs. 988 ± 176 ml, *P *= 0.05). End-inspiratory lung volume, including FRC (425 ± 66 ml), was slightly higher than estimated TLC (1512 ± 229 vs. 1413 ± 225 ml, *P *= 0.05).

**Figure 2:**
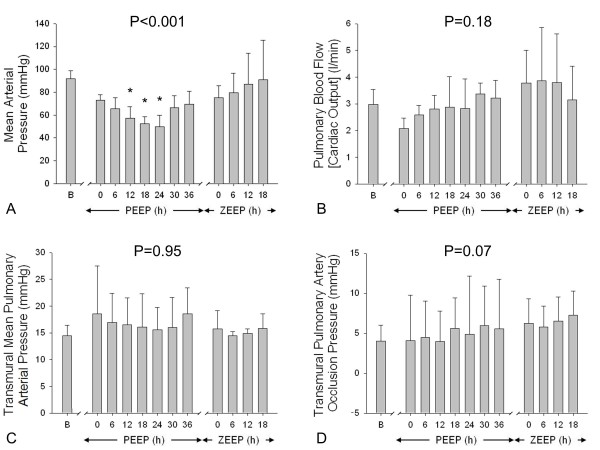
**Haemodynamics**. Mean arterial pressure (**panel A**, *n *= 5), cardiac output (**panel B**, *n *= 5), transmural pulmonary arterial pressure (**panel C**, *n *= 4) and transmural pulmonary artery occlusion pressure (**panel D**, *n *= 4) were recorded at baseline (B), during ventilation with high PEEP and low V_T _(36 h) and during ventilation with no PEEP (ZEEP) and low V_T _(18 h). During ventilation with high PEEP, oesophageal pressure at 0 cmH_2_O of airway pressure was assumed to have changed linearly from the value recorded at B (at 0 cmH_2_O) to the value recorded at time 0 of the ZEEP phase. *P *values refer to one-way RM ANOVA (on ranks if appropriate). *P <0.05 vs. B (Holm-Sidak or Dunn's method). B, baseline; PEEP, positive end-expiratory pressure; RM ANOVA, repeated measures analysis of variance; V_T_, tidal volume; ZEEP, zero end-expiratory pressure.

Changes in systemic and pulmonary haemodynamics are shown in Figure 2. Mean arterial pressure (and cardiac output) diminished soon after the application of PEEP despite aggressive fluid resuscitation and norepinephrine infusion (Additional file 4), but returned to baseline before PEEP was removed. Transmural mean pulmonary arterial pressure did not change over time while transmural pulmonary artery occlusion pressure tended to be higher than pre-study values following PEEP removal. By the end of the experiment, water balance ranged between +1600 and +6400 ml and body weight had accordingly increased (from 21 ± 1 to 25 ± 2 kg, *P *= 0.01).

Respiratory system mechanics, lung mechanics and gas exchange did not deteriorate during the PEEP phase (Additional file 5). Nonetheless, data recorded immediately after PEEP removal were slightly worse than those collected at baseline; as far as subsequent oedema formation was concerned, no further change occurred during the ZEEP phase (Table 1).

**Table 1:** Lung function

	Baseline	Ventilation with no PEEP (ZEEP phase)				*P*
		
		0 h	6 h	12 h	18 h	
End-inspiratory airway pressure (cmH_2_O)	10 ± 1	14 ± 3*	13 ± 2*	13 ± 3*	13 ± 3*	0.002

Transpulmonary pressure (cmH_2_O)	5 ± 2	10 ± 3*	9 ± 3*	9 ± 3*	10 ± 4*	0.001

Arterial oxygen tension (mmHg)	222 ± 43	187 ± 58	181 ± 44	180 ± 47	186 ± 24	0.11

Arterial carbon dioxide tension (mmHg)	41 ± 3	36 ± 4	39 ± 4	38 ± 3	39 ± 3	0.16

Respiratory system mechanics, lung mechanics and gas exchange at baseline and during ventilation with no PEEP (ZEEP phase) (18 h). End-inspiratory airway pressure was recorded during a 5-sec end-inspiratory pause. *P *values refer to one-way RM ANOVA (on ranks if appropriate). *P <0.05 vs. baseline (Holm-Sidak or Dunn's method). PEEP, positive end-expiratory pressure; ZEEP, zero end-expiratory pressure; RM ANOVA, repeated measures analysis of variance.

**Figure 3:**
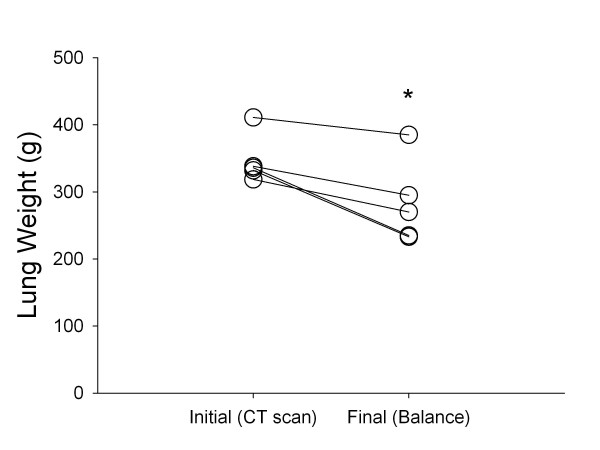
**Lung weight**. Individual changes in lung weight across the entire study period. Straight lines are used to connect individual points (not to suggest that changes occurred linearly over time). Initial lung weights were measured by quantitative analysis of lung CT obtained at baseline; final lung weights were measured on a balance following autopsy. *P = 0.01 (Student's paired *t *test). CT, computed tomography.

At autopsy, lungs looked pink and normally inflated, except for small areas of atelectasis (Additional file 6). Final lung weights did not exceed initial ones (Figure 3). Lung wet-to-dry weight ratio (5.1 ± 0.3 vs. 4.8 ± 0.7, *P *= 0.38) and histology (Table 2) did not differ from those of controls.

**Table 2:** Lung histology

	Present study	Previous study	*P*
Emphysematous changes	2 (2-3)	2 (2-3)	1.00

Interstitial congestion	2 (1-2)	2 (1-2)	0.23

Alveolar haemorrhage	1 (1-1)	1 (0-1)	0.60

Alveolar neutrophil infiltration	2 (2-2)	2 (1-3)	1.00

Alveolar macrophage proliferation	3 (2-3)	2 (1-2)	0.23

Alveolar type II pneumocytes proliferation	2 (2-2)	2 (2-2)	0.95

Interstitial lymphocytes proliferation	2 (1-2)	2 (1-2)	0.82

Interstitial thickening	2 (1-2)	2 (2-3)	0.05

Hyaline membranes formation	0 (0-0)	0 (0-1)	0.17

Interstitial fibrosis	1 (1-1)	2 (1-3)	0.02

Organization of alveolar exudate	1 (1-2)	1 (0-1)	0.19

Total score	18 (14-18)	18 (16-19)	0.43

Results of animals ventilated with high PEEP and low V_T _for 36 h and with no PEEP and low V_T _for 18 h (*n *= 5) (present study) were compared with those of animals ventilated with no PEEP and low V_T _for 54 h (*n *= 12) (previous study) [3]. Alterations were graded from 0 to 4, with higher scores indicating more severe and diffuse abnormalities. Total score was the sum of all individual scores. *P *values refer to Mann-Whitney rank sum test. PEEP, positive end-expiratory pressure; V_T_, tidal volume.

## Discussion

Healthy piglets ventilated with high PEEP and low V_T _for 36 h do not develop pulmonary oedema when airway pressure is suddenly lowered (by removing PEEP). This proves that high PEEP and low V_T _do not permanently alter the integrity of the alveolar-capillary interface even if lung inflation approaches its upper physiological limit (volume of gas globally inflated equal to IC).

We have previously shown that healthy piglets ventilated for 54 h with high PEEP (never removed) and low V_T _survive with normal lungs [4]. There, lack of pulmonary oedema could have been attributed to diminished transmural pulmonary capillary pressure (that drives fluid extravasation) across an abnormally highly permeable (overstretched) blood-gas barrier. In fact, application of high PEEP always caused hypotension and low cardiac output while lungs were maximally inflated [4].

PEEP may or may not prevent lung oedema (or even worsen it) depending on relative changes in pulmonary intravascular and extravascular (alveolar and extra-alveolar) pressures, lung surface area (through which filtration occurs), pulmonary endothelium and epithelium porosity [16-20]. Since its initial use, PEEP was mainly thought to tamponade great veins (thus lowering pulmonary blood flow and pressure) and 'exert an opposing force tending to hinder the outpouring of both red corpuscles and serum from the pulmonary capillaries' [21]. Other means of lowering intravascular pulmonary pressure, such as phlebotomy or vasodilatation, can attenuate pulmonary oedema formation, even if the blood-gas barrier is abnormally permeable [22,23]. On the other side, increasing transmural pulmonary capillary pressure with dopamine [8] or fluids [24-26] aggravates inflammatory pulmonary oedema and 'sudden removal of a previously existing backward pressure on the pulmonary capillaries (that is PEEP) is followed by an increased permeability of the capillary wall' and leakage of serum [21]. Therefore, lack of oedema *per se *does not prove that high PEEP and low V_T _are safe.

In this present work, we specifically addressed one of the mechanisms possibly underlying our [4] and others' [6,8] previous findings (namely, diminished pulmonary capillary filtration pressure) by abruptly removing a potential obstacle to oedema formation (high PEEP itself). We ventilated healthy piglets with high PEEP and low V_T _(volume of gas globally inflated around estimated IC) for 36 h. By this time, animals ventilated with large V_T _alone (always resulting in the same end-inspiratory lung volume) develop overt pulmonary oedema, as indicated by heavy and congested lungs, altered respiratory system mechanics and gas exchange, and pulmonary and systemic inflammation [3]. Since lung injury may not become macroscopically evident as long as PEEP counteracts fluid filtration, PEEP was then suddenly zeroed. Fluid filtration should have freely occurred, driven by normal (and occasionally supra-normal) haemodynamics, had the blood-gas barrier been disrupted [18,24,27].

Acting similarly, we have previously demonstrated a small, non-significant increase in lung weight following PEEP removal in four healthy piglets, finally ventilated with low V_T _for 3 h [4]. In order to exclude that overt pulmonary oedema could have developed had the experiments lasted longer, we decided to extend the duration of ventilation with low V_T _and no PEEP to 18 h. Even so, lung weight did not increase (and it actually decreased, possibly as a consequence of exsanguination [28]) and mechanics and gas exchange did not deteriorate with time. This proves that ventilation with high PEEP and low V_T _over the previous 36 h had not grossly and permanently altered the permeability of the blood-gas barrier. The minor decrease in pulmonary compliance and arterial oxygenation that became evident when PEEP was removed (and did not worsen over time) was possibly related to largely positive water balance (increased pulmonary blood volume) and non-specific alterations in lung histology. Of note, in our setting, pulmonary oedema is always a clear-cut diagnosis: lung weight increases by 300 to 600 g, respiratory system and lung compliance largely and progressively decrease, and hypoxemia and hypercapnia are always severe [3,4]. None of these changes occurred in this present series of animals.

Reasons why high PEEP and low V_T _do not cause pulmonary oedema, despite resulting in extremely large lung inflation, are not known. On one side, avoidance of large V_T _(and high inspiratory flows) may have a major role as lungs behave as viscoelastic bodies that will fail if elongated too much (and too rapidly) [4,29]. On the other side, high PEEP may have a direct protective effect, diminishing inherent pulmonary heterogeneity and local stress amplification [30].

Our present and past [4] results support maximal lung recruitment and minimal tidal ventilation, as during low-frequency positive-pressure ventilation with extracorporeal carbon dioxide removal [31]. However, caution is advised in translating data from pre-clinical experience to humans with injured lungs. For instance, another strategy based on the same sound rationale − high-frequency oscillatory ventilation − recently failed to benefit patients with acute respiratory distress syndrome [32,33], despite promising pre-clinical results.

Some limitations of this study deserve a comment. First, only five animals were enrolled because, even in such a small group, results were highly consistent and reproducible. Second, haemodynamic monitoring did not include echocardiography. Although some degree of tricuspid regurgitation may have occurred during the first part of the study (undermining cardiac output measurement), this likely reversed once PEEP had been removed. By that time, not only cardiac output but several other haemodynamic variables (for instance, blood pressure, diuresis, urinary electrolytes, arteriovenous oxygen difference and lactatemia) were normal (or even above normal). The bulk of the data strongly suggest that low cardiac output was not an issue, at least during the second part of the study. Third, integrity and permeability of the blood-gas barrier were not directly assessed and minor changes cannot be completely excluded; but still, pulmonary oedema, *de facto*, never occurred. Finally, lung injury may have rapidly reversed once overinflation had been suddenly removed [22]; however, lowering mean airway pressure once the blood-gas barrier has been damaged usually causes alveolar flooding, with precipitous deterioration of lung mechanics and gas exchange [27].

## Conclusions

Ventilation with high PEEP and low V_T _does not cause oedema in healthy lungs, not even after PEEP has been suddenly removed and pulmonary haemodynamics have returned to normal. This suggests that large, but mainly static, lung inflation does not permanently alter the integrity and permeability of the blood-gas barrier.

## Key messages

• Healthy lungs ventilated with high PEEP and low V_T _do not develop pulmonary oedema, even if globally inflated up to their total capacity.

• High PEEP (and low V_T_) do not merely hydrostatically counteract pulmonary fluid extravasation.

• Mechanical ventilation with high PEEP and low V_T _does not grossly injure the blood-gas barrier in healthy lungs.

## Abbreviations

CT: computed tomography; FRC: functional residual capacity; IC: inspiratory capacity; PEEP: positive end-expiratory pressure; RM ANOVA: repeated measures analysis of variance; TLC: total lung capacity; V_T: _tidal volume; ZEEP: zero end-expiratory pressure.

## Competing interests

This study was funded in part by GE Healthcare. The authors declare that they have no competing interests that can have influenced the submitted manuscript.

## Authors' contributions

AP participated in the study design, statistical analysis and wrote the manuscript. DTA participated in the study design, ran the experiments, performed statistical analysis and revised the manuscript. GEI participated in the study design, ran the experiments, performed statistical analysis and revised the manuscript. MM participated in the study design and data acquisition. BC participated in data acquisition and analysis.MM participated in data acquisition and analysis. LZ participated in data acquisition and analysis. SG performed the surgical preparation and revised the manuscript. LL performed the lung computed tomography and participated in the study design. LG participated in the study design and revised the manuscript. All authors read and approved the final version of the manuscript.

## Supplementary Material

Additional file 1 **Bland-Altman plot of lung weight measured with balance vs. CT scan**. Five other healthy piglets (18 ± 1 kg) underwent lung CT at 0 cmH_2_O of airway pressure and were then sacrificed and exsanguinated. Results of quantitative analysis of CT scan were compared with excised lung weight, measured on a balance. The Bland-Altman plot of lung weight measured with the two techniques show a bias -91 g, limits of agreement -128 - -54 g). Of note, in piglets of similar weight, pulmonary blood volume should be around 90 ml (see [28]) CT, computed tomography. Click here for file

Additional file 2 **Additional methods**. Further details on surgical preparation, quantitative analysis of lung CT, haemodynamic protocol, sacrifice and autopsy are shown. CT, computed tomography. Click here for file

Additional file 3 **Baseline lung CT**. Results of quantitative analysis of lung CT scans, which allows for exact computation of lung volumes and capacities and volume of gas due to PEEP. CT, computed tomography; PEEP, positive end-expiratory pressure. Click here for file

Additional file 4 **Additional haemodynamic variables throughout the experiment**. Urinary output (**panel A**, *n *= 5), water balance (**panel B**, *n *= 5), urinary electrolytes (**panel C and D**, *n *= 4), arteriovenous oxygen difference (**panel E**, *n *= 5), blood lactate (**panel F**, *n *= 5), and rate of norepinephrine infusion (**panel G**, *n *= 5) were recorded at baseline (B), during ventilation with high PEEP and low V_T _(36 h) and during ventilation with no PEEP (ZEEP) and low V_T _(18 h). *P *values refer to one-way RM ANOVA (on ranks if appropriate); *P <0.05 vs. B (Holm-Sidak or Dunn's method). B, baseline; PEEP, positive end-expiratory pressure; RM ANOVA, repeated measures analysis of variance; V_T_, tidal volume; ZEEP, zero end-expiratory pressure. Click here for file

Additional file 5 **Lung function during ventilation with high PEEP and low V_T_**. Respiratory system mechanics (**panel A**) lung mechanics (**panel B**), and gas exchange (**panel C and D**) were recorded during 36 h of ventilation with high PEEP and low V_T_. Oesophageal pressure at 0 cmH_2_O of airway pressure was assumed to have changed linearly from the value recorded at baseline (B) (at 0 cmH_2_O) to the value recorded at time 0 of the ZEEP phase. *P *values refer to one-way RM ANOVA (on ranks if appropriate); *P <0.05 vs. B (Holm-Sidak or Dunn's method). B, baseline; PEEP, positive end-expiratory pressure; RM ANOVA, repeated measures analysis of variance; V_T_, tidal volume; ZEEP, zero end-expiratory pressure. Click here for file

Additional file 6 **Autoptic lung appearance**. Lungs of animals ventilated with high PEEP and low V_T _for 36 h and with no PEEP and low V_T _for 18 h (present study) are shown in **panel A**. For comparison, lungs of animals ventilated for 54 h with no PEEP and low V_T _(lung weight changed from 377 to 220 g) (**panel B**), with no PEEP and large V_T _(close to inspiratory capacity) (lung weight increased from 395 to 721 g) (**panel C**) and with high PEEP and low V_T _for 54 h (lung weight changed from 282 to 290 g) (**panel D**) are shown. PEEP, positive end-expiratory pressure; V_T_, tidal volume. Click here for file
