# Supplementary figures and images for: High positive end-expiratory pressure: only a dam against oedema formation?
Source: Crit Care. 2013 Jul 11;17(4):R131. doi: 10.1186/cc12810 (PMC4056428; doi:10.1186/cc12810)

## Slide 1
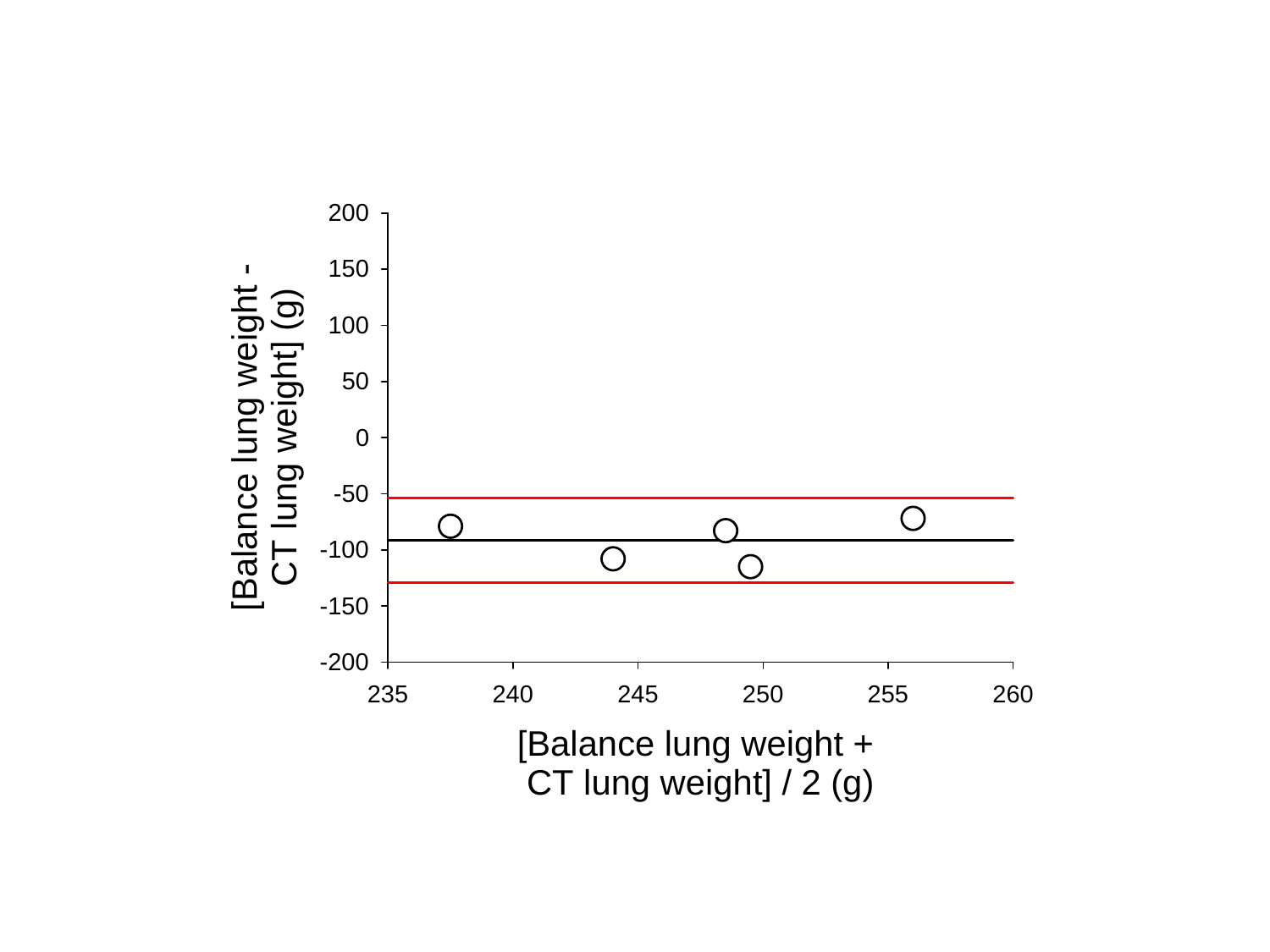

Supplement: Additional file 1 — Bland-Altman plot of lung weight measured with balance vs. CT scan. Five other healthy piglets (18 ± 1 kg) underwent lung CT at 0 cmH2O of airway pressure and were then sacrificed and exsanguinated. Results of quantitative analysis of CT scan were compared with excised lung weight, measured on a balance. The Bland-Altman plot of lung weight measured with the two techniques show a bias -91 g, limits of agreement -128 - -54 g). Of note, in piglets of similar weight, pulmonary blood volume should be around 90 ml (see [28]) CT, computed tomography. [file cc12810-S1.PPT]

## Slide 1
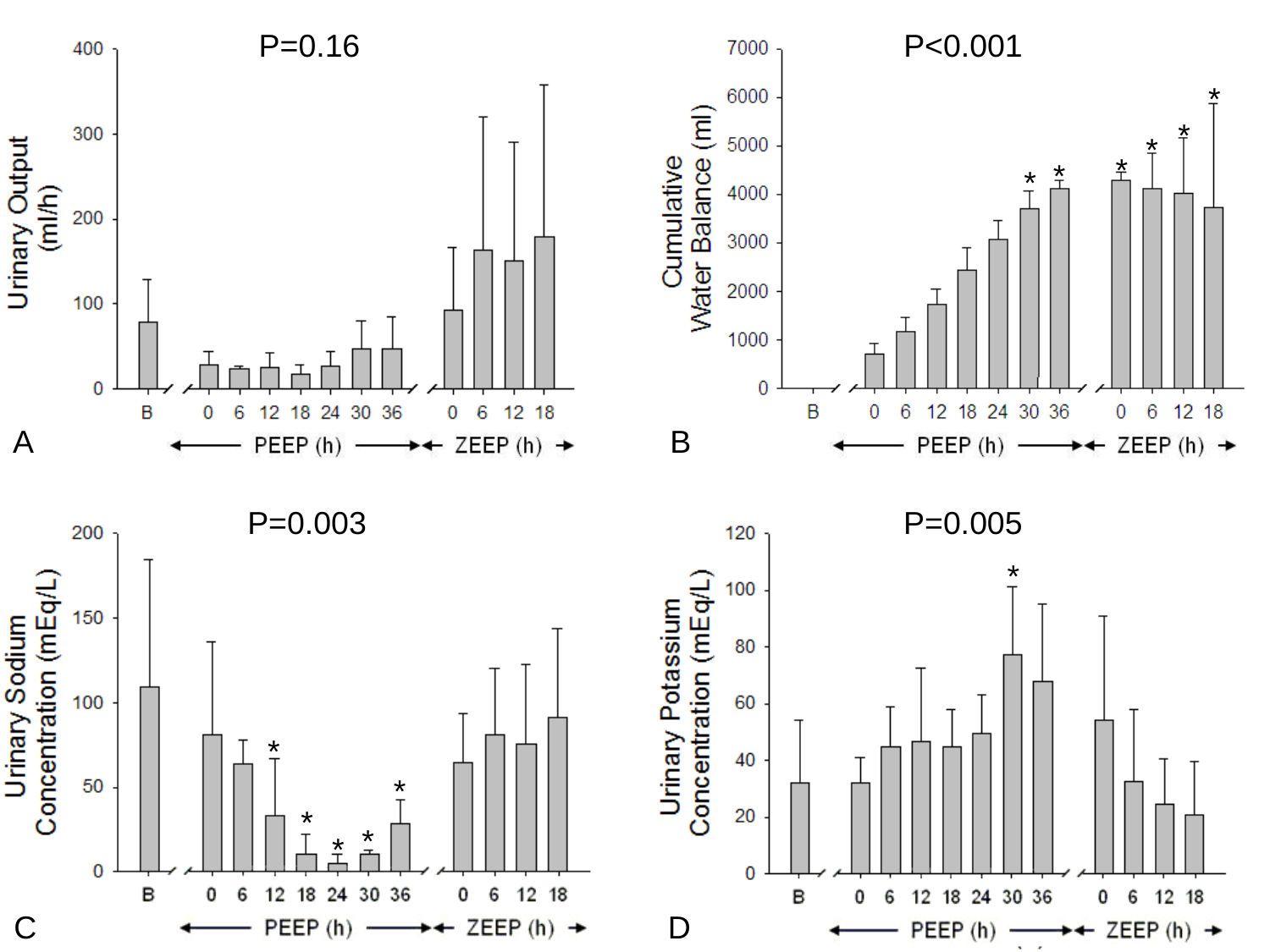

P=0.16
P<0.001
*
*
*
*
*
*
A
B
P=0.003
P=0.005
*
*
*
*
*
*
C
D

## Slide 2
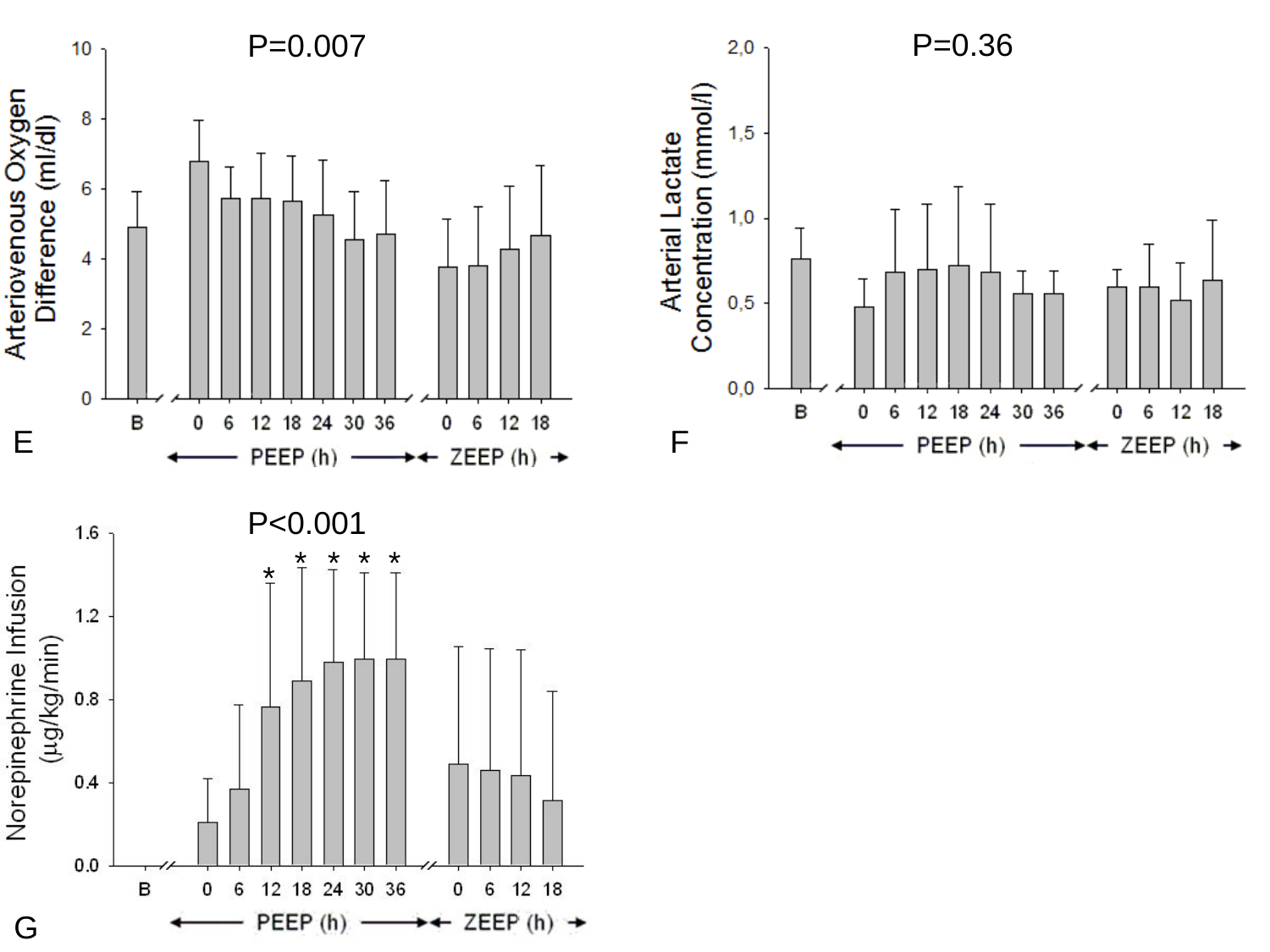

P=0.36
P=0.007
E
F
P<0.001
*
*
*
*
*
G

Supplement: Additional file 4 — Additional haemodynamic variables throughout the experiment. Urinary output (panel A, n = 5), water balance (panel B, n = 5), urinary electrolytes (panel C and D, n = 4), arteriovenous oxygen difference (panel E, n = 5), blood lactate (panel F, n = 5), and rate of norepinephrine infusion (panel G, n = 5) were recorded at baseline (B), during ventilation with high PEEP and low VT (36 h) and during ventilation with no PEEP (ZEEP) and low VT (18 h). P values refer to one-way RM ANOVA (on ranks if appropriate); *P <0.05 vs. B (Holm-Sidak or Dunn's method). B, baseline; PEEP, positive end-expiratory pressure; RM ANOVA, repeated measures analysis of variance; VT, tidal volume; ZEEP, zero end-expiratory pressure. [file cc12810-S4.PPT]

## Slide 1
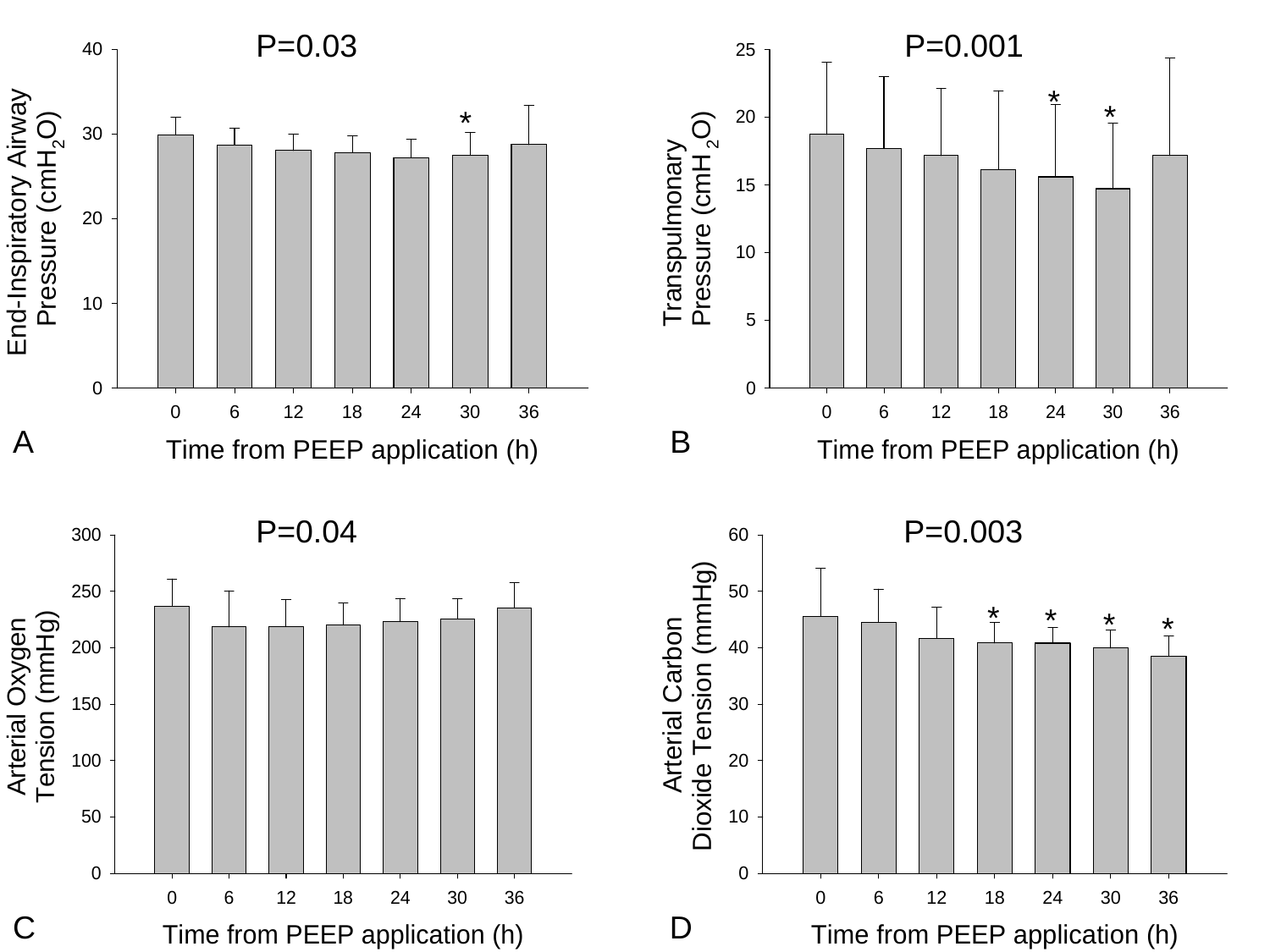

P=0.03
P=0.001
*
*
*
A
B
P=0.04
P=0.003
*
*
*
*
C
D

Supplement: Additional file 5 — Lung function during ventilation with high PEEP and low VT. Respiratory system mechanics (panel A) lung mechanics (panel B), and gas exchange (panel C and D) were recorded during 36 h of ventilation with high PEEP and low VT. Oesophageal pressure at 0 cmH2O of airway pressure was assumed to have changed linearly from the value recorded at baseline (B) (at 0 cmH2O) to the value recorded at time 0 of the ZEEP phase. P values refer to one-way RM ANOVA (on ranks if appropriate); *P <0.05 vs. B (Holm-Sidak or Dunn's method). B, baseline; PEEP, positive end-expiratory pressure; RM ANOVA, repeated measures analysis of variance; VT, tidal volume; ZEEP, zero end-expiratory pressure. [file cc12810-S5.PPT]

## Slide 1
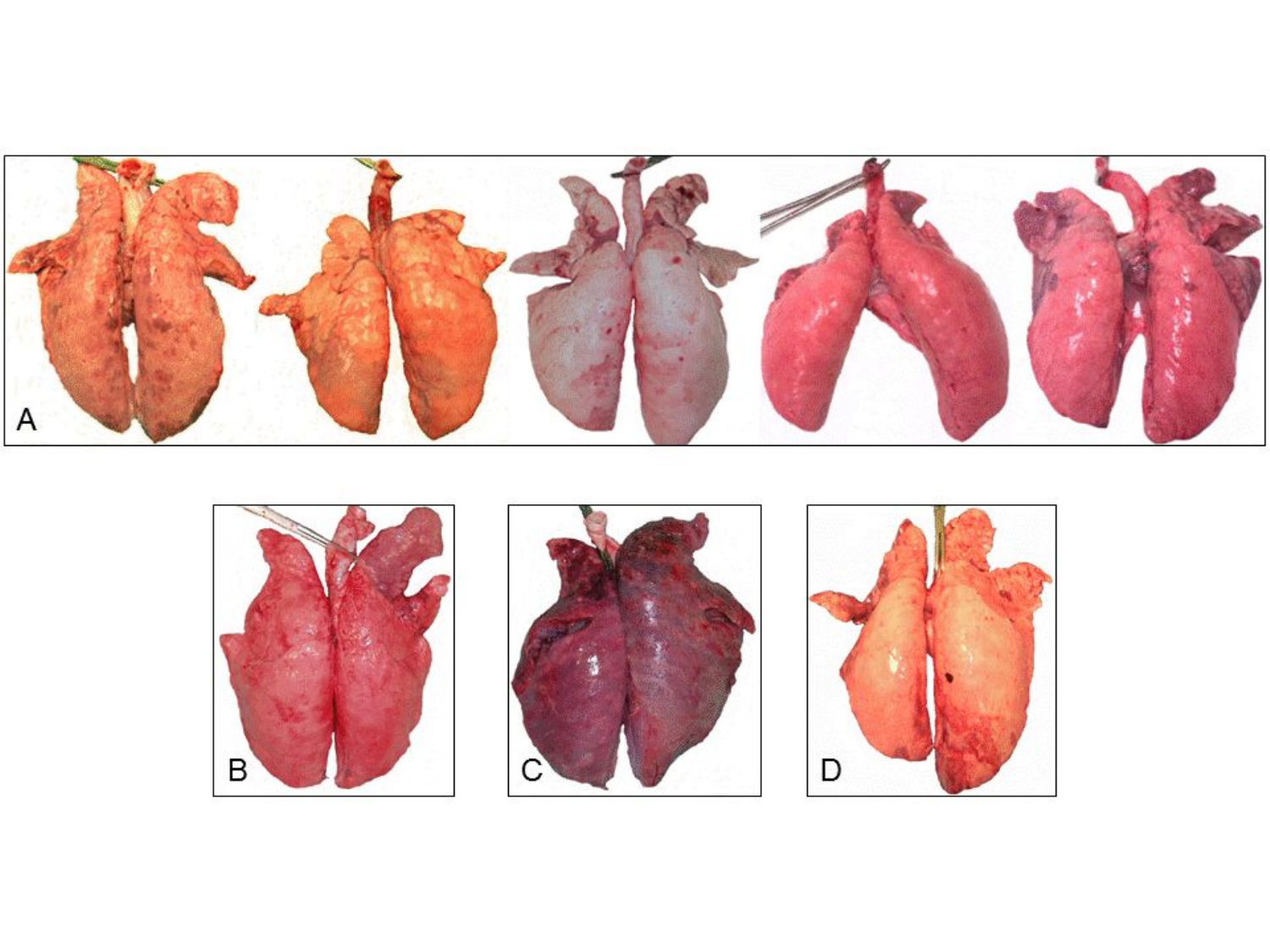

Supplement: Additional file 6 — Autoptic lung appearance. Lungs of animals ventilated with high PEEP and low VT for 36 h and with no PEEP and low VT for 18 h (present study) are shown in panel A. For comparison, lungs of animals ventilated for 54 h with no PEEP and low VT (lung weight changed from 377 to 220 g) (panel B), with no PEEP and large VT (close to inspiratory capacity) (lung weight increased from 395 to 721 g) (panel C) and with high PEEP and low VT for 54 h (lung weight changed from 282 to 290 g) (panel D) are shown. PEEP, positive end-expiratory pressure; VT, tidal volume. [file cc12810-S6.PPT]
