# Supplementary material for: High positive end-expiratory pressure: only a dam against oedema formation?
Source: Crit Care. 2013 Jul 11;17(4):R131. doi: 10.1186/cc12810 (PMC4056428; doi:10.1186/cc12810)
Supplement: Additional file 2 — Additional methods. Further details on surgical preparation, quantitative analysis of lung CT, haemodynamic protocol, sacrifice and autopsy are shown. CT, computed tomography. [file cc12810-S2.PDF]

Additional methods

Surgical preparation

Anaesthesia was induced with medetomidine (0.025 mg/kg IM) and tiletamine/zolazepam (5 mg/kg IM). An auricular vein was cannulated and propofol was administered (2 mg/kg bolus dose, then 5 mg/kg/h IV). Surgical preparation was then carried out under sterile condition. Tracheotomy was performed after lidocaine infiltration. Mechanical ventilation was started with V_T 10 ml/kg of body weight, 15 breaths per minute, inspiratory-to-expiratory time ratio 1:2, no externally applied PEEP, inspired oxygen fraction 0.50. Right carotid artery was surgically cannulated. A three lumen central venous and a pulmonary artery catheter were inserted through the right internal jugular vein and advanced under waveform guidance. A bladder catheter was positioned via cistostomy. A latex, thin-walled, 5-cm long, oesophageal balloon was advanced into the inferior third of the oesophagus and filled with 1.5 ml of room air. Proper positioning of endovascular catheters and oesophageal balloon was verified on lung CT and confirmed at autopsy. Pressure transducers were connected to the endotracheal tube, the oesophageal balloon and the endovascular catheters, zeroed at room air or heart level, as appropriate. Data were recorded and analyzed using a dedicated software (Colligo, www.elekton.it). Urinary electrolytes were measured with a newly developed urine analyser (Kidney Instant MonitorinG, K.IN.G., Orvim, Milano) [1]. Anaesthesia was maintained with propofol (5-10 mg/kg/h), pancuronium bromide (0.3-0.5 mg/kg/h) and medetomidine (2.5-10.0 μg/kg/h). Normal saline was administered at 100 ml/h during surgery and 50 ml/h thereafter, unless otherwise indicated by haemodynamic requirements (see below). Ceftriaxone (1g IV) and

tramadol (50 mg IV) were administered preoperatively and every 12 h. Low-molecular-weight heparin (2000 IU) was given subcutaneously once a day.

Quantitative analysis of lung CT

Lung profiles were manually delineated on each slice and analysis performed with a dedicated software (SoftEFilm, www.elekton.it). Lung tissue was classified as: non-aerated (density > -100 HU), poorly-aerated (density between -100 and -500 HU), well-aerated (density between -500 and -900 HU) or over-aerated (density < -900 HU) [2].

Haemodynamic protocol

Following surgical preparation, normal saline (NaCl 0.9%) was infused at 50 ml/h. A bolus of 250 ml was infused immediately before the application of high PEEP. Thereafter, saline and norepinephrine use was protocolised to maintain mean arterial pressure above 60 mmHg. If mean arterial pressure was below 60 mmHg for at least 30 min, the following actions were sequentially performed:

1. Saline 100 ml bolus, then restart infusion at 50 ml/h
2. Saline 150 ml bolus, then increase infusion at 75 ml/h
3. Norepinephrine 0.14 – 0.28 μg/kg/min, keep saline infusion at 75 ml/h
4. Saline 150 ml bolus, then increase infusion at 100 ml/h
5. Norepinephrine 0.35 - 1.4 μg/kg/min, keep saline infusion at 100 ml/h

If at the maximal rate of saline and norepinephrine use mean arterial pressure was still below 60 mmHg, hypotension was tolerated and no other intervention was made. When mean arterial pressure rose above 70 mmHg, haemodynamic support was de-escalated according to the same protocol.

Sacrifice and autopsy

Pigs were sacrificed with a bolus of KCl (40 mEq IV) under deep sedation. The tracheal tube was clamped and the animals weighted. The chest wall was then opened and the animals were exsanguinated. The trachea was cut and the lungs excised; the main bronchi were clamped, cut at the hilum and the lungs were separately weighted. Five tissue samples were randomly taken from the right lung, weighted (wet weight), dried for 24 h at 50 °C and weighted again (dry weight). Individual mean wet-to-dry weight ratios were computed.

References

1. Caironi P, Langer T, Taccone P, Bruzzone P, De Chiara S, Vagginelli F, Caspani L, Marenghi C, Gattinoni L: **Kidney instant monitoring (K.IN.G®): a new analyser to monitor kidney function**. *Minerva Anestesiol* 2010; **76**:316-324.

2. Gattinoni L, Caironi P, Pelosi P, Goodman LR: **What has computed tomography taught us about the acute respiratory distress syndrome?** *Am J Respir Crit Care Med* 2001; **164:**1701-1711.
