# Supplementary material for: High positive end-expiratory pressure: only a dam against oedema formation?
Source: Crit Care. 2013 Jul 11;17(4):R131. doi: 10.1186/cc12810 (PMC4056428; doi:10.1186/cc12810)
Supplement: Additional file 3 — Baseline lung CT. Results of quantitative analysis of lung CT scans, which allows for exact computation of lung volumes and capacities and volume of gas due to PEEP. CT, computed tomography; PEEP, positive end-expiratory pressure. [file cc12810-S3.PDF]

Baseline lung CT

	FRC (0 cmH_2O)	PEEP (~18 cmH_2O)	TLC (45 cmH_2O)	P
Gas volume (ml)	425±66	1299±230*	1413±225*	<0.01
Lung weight (g)	347±36	361±28	349±7	0.32
Non-aerated lung tissue (g)	15±5	16±5	16±2	0.87
Poorly-aerated lung tissue (g)	118±21	50±5*	50±9*	<0.01
Well-aerated lung tissue (g)	214±27	272±18*	292±24*	0.01
Over-inflated lung tissue (g)	0±0	2±2	11±9*$^{\#}$	0.02

Results of quantitative analysis of lung CT taken at baseline at three different airway pressures: functional residual capacity, PEEP, and total lung capacity. FRC: functional residual capacity; PEEP: positive end-expiratory pressure; TLC: total lung capacity; p values refer to one-way RM ANOVA (on ranks if appropriate); * p<0.05 *vs.* FRC; $^{\#}$ p<0.05 *vs.* PEEP (Holm-Sidak or Dunn's method).
